# Awareness of Emotional Stimuli Determines the Behavioral Consequences of Amygdala Activation and Amygdala-Prefrontal Connectivity

**DOI:** 10.1038/srep25826

**Published:** 2016-05-16

**Authors:** R. C. Lapate, B. Rokers, D. P. M. Tromp, N. S. Orfali, J. A. Oler, S. T. Doran, N. Adluru, A. L. Alexander, R. J. Davidson

**Affiliations:** 1 Department of Psychology, University of Wisconsin – Madison, 1500 Highland Avenue, Madison, Wisconsin 53705, USA; 2 Waisman Laboratory for Brain Imaging & Behavior, University of Wisconsin – Madison, 1500 Highland Avenue, Madison, Wisconsin 53705, USA; 3 Center for Healthy Minds, University of Wisconsin – Madison, 1500 Highland Avenue, Madison, Wisconsin 53705, USA; 4 Department of Psychology, Utrecht University, Heidelberglaan 2, 3584 CS, Utrecht, the Netherlands; 5 Department of Psychiatry, University of Wisconsin – Madison, 6001 Research Park Boulevard, Madison, Wisconsin 53719, USA; 6 Department of Medical Physics, University of Wisconsin – Madison, 1500 Highland Avenue, Madison, Wisconsin 53705, USA

## Abstract

Conscious awareness of negative cues is thought to enhance emotion-regulatory capacity, but the neural mechanisms underlying this effect are unknown. Using continuous flash suppression (CFS) in the MRI scanner, we manipulated visual awareness of fearful faces during an affect misattribution paradigm, in which preferences for neutral objects can be biased by the valence of a previously presented stimulus. The amygdala responded to fearful faces independently of awareness. However, when awareness of fearful faces was prevented, individuals with greater amygdala responses displayed a negative bias toward unrelated novel neutral faces. In contrast, during the aware condition, inverse coupling between the amygdala and prefrontal cortex reduced this bias, particularly among individuals with higher structural connectivity in the major white matter pathway connecting the prefrontal cortex and amygdala. Collectively, these results indicate that awareness promotes the function of a critical emotion-regulatory network targeting the amygdala, providing a mechanistic account for the role of awareness in emotion regulation.

Consciously aware processing—processing accompanied by subjective experience and reportable under normal circumstances^1^,^2^—is but a fragment of what shapes our physiology and behavior. For example, negative facial expressions can increase activation of the amygdala^3^,^4^, alter peripheral physiology^5^,^6^,^7^, and influence judgments^8^,^9^, even when they are processed outside of conscious awareness. In the clinic, automatic emotional processing is regarded as maladaptive, and increasing awareness of emotional triggers to ameliorate the symptoms of affective disorders is a common goal across distinct therapeutic approaches^10^,^11^,^12^. Despite the widespread idea that awareness may benefit emotion regulation, whether the function of emotion-regulatory circuitry is promoted by conscious awareness of emotional stimuli remains unknown. Here, we show both functional and structural neuroimaging evidence that a critical amygdala-prefrontal emotion-regulatory network selectively impacts behavior following awareness of negative cues.

Findings from psychophysiological and neuroimaging studies have consistently highlighted the relatively “optional” nature of conscious awareness for the initial bottom-up processing of biologically relevant emotional stimuli. Specifically, early appraisal regions of the brain, such as the amygdala, are often engaged by facial expressions independently of awareness, such as when those expressions are rendered invisible via masking^3^,^4^,^13^,^14^, processed by the blind visual field of blindsight patients^15^,^16^ or by individuals exhibiting spatial neglect^17^. Such amygdala engagement may be significant if it includes its major output center, the central nucleus (CeA), which promotes the rapid generation of behavioral, endocrine, and autonomic responses^18^. Accordingly, fear-relevant stimuli processed outside of awareness can provoke changes across several peripheral-physiological channels indicative of autonomic nervous system engagement, including heart rate^19^, skin conductance^5^,^6^,^7^, and pupil dilation^20^. Collectively, these studies demonstrate that conscious awareness of certain emotional stimuli is not required for the mobilization of central and peripheral systems indicative of initial emotional stimulus encoding and reactivity. But is awareness of these emotional stimuli epiphenomenal, or does it serve any utility for human behavior?

Recent data suggest that awareness may serve an emotion-regulatory role by preventing the initial “bottom-up” reactivity to an emotional stimulus from automatically biasing evaluative behavior. Specifically, we have demonstrated that when fearful faces are processed without visual awareness, skin conductance responses to them are associated with a greater dislike of novel neutral faces presented subsequently^7^. In contrast, when fearful faces are visible and consciously processed, skin conductance responses are uncorrelated with subsequent judgments. These findings resonate with predictions from Schwarz & Clore’s “affect-as-information” theory, which postulates that affect processed in the absence of awareness of its source is prone to bind to unrelated objects in the environment, resulting in greater “*affect misattribution*”^21^. Similarly, valence-congruent affective priming can be amplified when awareness of affective primes is prevented^19^,^22^,^23^,^24^. Upon investigating how awareness may reduce affect misattribution in healthy individuals, we found that rather than attenuating the initial bottom-up, physiological reactivity to fearful cues, awareness seems to “break” otherwise automatic associations between initial (physiological) reactions and subsequent evaluative behavior^7^.

The goal of the present study was to determine the neural mechanisms that selectively operate during *consciously aware* emotional processing to attenuate the influence of incidental and unrelated emotional stimuli on evaluative behavior. We refer to the misattribution or transfer of valence from an emotional stimulus onto a neutral one as “*affective coloring*”. We hypothesized that affective coloring behavior may be differentially associated with “bottom-up” initial emotional-stimulus encoding versus “top-down” (possibly emotion-regulatory) neural mechanisms, depending on awareness. Specifically, as fearful faces, even if masked, often increase activation of the amygdala^4^,^25^, we hypothesized that the magnitude of affective coloring following unaware fearful-face processing would covary with initial amygdala responses.

Conversely, given the predicted emotion-regulatory role for conscious awareness, and that awareness is often associated with increased prefrontal cortical (PFC) engagement^1^, we hypothesized that PFC circuitry known to support emotion regulation, including functional and structural connectivity between the amygdala and the PFC, would be implicated in the reduction of affective coloring following aware processing^26^,^27^,^28^. During successful emotion regulation, the amygdala interacts with particular sectors of the PFC^29^,^30^, such as ventromedial and mid-lateral, typically manifesting as negative or inverse (putatively inhibitory) coupling between these regions^26^,^27^,^28^,^31^,^32^. We therefore sought to examine whether the behavioral function of this amygdala-PFC functional circuitry is enhanced by emotional-stimulus awareness, as indicated by an association between inverse amygdala-prefrontal coupling and reduced affective coloring specifically following aware fearful-face processing.

As a convergent investigation of this idea, we also examined individual differences in trait-like amygdala-prefrontal structural connectivity. The amygdala is heavily connected to the PFC primarily via a white matter tract called the uncinate fasciculus. Greater structural connectivity in the uncinate, as measured with diffusion imaging, has been typically associated with more favorable emotion-regulatory outcomes^27^,^33^,^34^. We posited that if function of this amygdala-prefrontal circuitry is enhanced by awareness of emotional stimuli, a more robust amygdala-prefrontal structural connection would be associated with reduced affective coloring behavior following aware (compared to unaware) fearful-face processing.

To test these hypotheses, we took our previously developed affect misattribution paradigm^7^ into the MRI scanner, where we manipulated awareness of visual stimuli within-subjects using continuous flash suppression (CFS^35^), a powerful method based on binocular rivalry (Fig. 1). In CFS, a colorful pattern flashes to one eye at ~10 Hz, and a low-contrast static stimulus is presented to the other eye. The participant subjectively perceives the flashing pattern while the static stimulus can remain suppressed from awareness for long durations (e.g., ~1000–5000 ms). Fearful faces and flowers (matched for luminance and contrast) were used as the negative and neutral stimuli, respectively. The use of non-social shapes (e.g., flowers) as the control stimuli is consistent with our prior psychophysiological investigation that unveiled dissociations between aware and unaware emotional processing^7^, and is also consistent with methods used in prior studies from other laboratories^36^,^37^,^38^,^39^ since it avoids limitations associated with potentially ambiguous and amygdala-engaging stimuli (such as neutral faces)^40^,^41^.

To index affective coloring, individuals rated the likeability of novel neutral faces shown on average 7 s after the fearful faces and flowers. Affective coloring was operationalized as lower likeability ratings for novel faces presented after fearful faces (compared with flowers). Given considerable^7^ and stable^42^ individual differences in the magnitude of affective coloring behavior following emotional processing, we capitalized on this inter-individual heterogeneity to examine neural mechanisms selectively associated with affective coloring in aware and unaware processing conditions. Specifically, we examined the associations between affective coloring, amygdala responses, and functional and structural amygdala-PFC connectivity across individuals, and tested whether awareness modulated those associations.

## Results

### Likeability Ratings

We explored whether likeability ratings of neutral faces were on average modulated by valence or awareness, and we found that those factors were not significant, fearful faces vs. flowers; *F*(1,30) = 0.38, *p* > 0.5; aware vs. unaware (*F*(1,30) = 0.01, *p* > 0.9). These results underscore the large inter-individual heterogeneity in affective coloring behavior^7^, which was previously shown to be stable^42^—thus justifying the investigation of the neural bases of affective coloring behavior using individual differences analysis.

### Aware stimulus processing is associated with increased prefrontal cortical engagement

Replicating prior findings (for a review, see^1^), we found that when individuals were aware of the stimuli, regions of the frontoparietal network, including lateral and dorsomedial prefrontal cortex were significantly more engaged compared to when individuals were unaware (Table 1).

### Fearful faces increase amygdala BOLD independently of visual awareness, but only unaware amygdala activation is associated with subsequent affective coloring

We examined how fearful faces modulated responses in a previously reported region of interest focused on the central nucleus of the amygdala (CeA), a primary recipient of stimulus-value computations from the basal and lateral amygdala nuclei, and the major amygdala output center that rapidly mobilizes peripheral-physiological responses^18^,^43^,^44^. Extending prior studies suggesting that the amygdala does not require conscious awareness to respond to facial expressions^3^,^4^,^45^, BOLD signal in the right CeA increased to fearful faces (relative to flowers) equivalently in both visually aware and unaware conditions (Fig. 2A).

We next examined whether amygdala responses were associated with likeability ratings. We found that greater right amygdala responses during unaware fearful-face processing were associated with lower liking of later-presented novel neutral faces (ρ (Spearman’s rho) = −0.40, *p* < 0.05) (Fig. 2B). In contrast, amygdala responses during aware processing were not reliably associated with subsequent ratings of neutral faces (and instead tended to be associated with greater, rather than lower preferences for those faces, ρ = 0.32, *p* = 0.076). A significant difference in the slopes reflecting the relationship between amygdala responses [fearful faces – flowers] and neutral face likeability (shown after [fearful faces – flowers]) in the aware compared to the unaware condition demonstrated that awareness significantly impacted the association between amygdala responses and preference judgment behavior (95% CI_slope difference_: −1.13, −0.19; statistical significance at *p* < 0.05 is denoted by the interval not containing zero^46^). Therefore, when unchecked by conscious awareness, greater amygdala responses to fear cues were associated with a negative bias towards later-shown neutral stimuli—i.e., greater affective coloring.

### Reduced affective coloring following aware processing of negative stimuli is associated with the inverse coupling between the amygdala and lateral and dorsomedial PFC

Next, we examined whether function of prefrontal circuitry was associated with a reduction of affective coloring when individuals were aware of the stimuli. Given that PFC engagement increased with awareness, and that the inverse amygdala-PFC coupling has been associated with successful emotion regulation during (aware) emotion-processing tasks^26^,^27^,^28^,^31^,^32^, we tested whether inverse amygdala-prefrontal coupling was associated with an attenuation of affective coloring behavior, and whether awareness strengthened that association. To do so, we conducted a functional connectivity analysis (psychophysiological interaction; PPI^47^) using the right CeA region as a seed, and ran a voxelwise regression of affective coloring on amygdala connectivity weights separately for aware and unaware conditions. We hypothesized that the inverse amygdala-PFC coupling would minimize affective coloring. Moreover, if conscious awareness promotes the function of this (putatively emotion-regulatory) neural mechanism, the association between amygdala-PFC coupling and behavior should be specific and selective to the consciously aware condition.

As shown on Fig. 3A and Table 2, when individuals were aware of fearful faces, less affective coloring was associated with the inverse coupling between the right amygdala and two PFC clusters: bilateral dorsomedial PFC (dmPFC), and left lateral PFC (lPFC, including dorsal and ventral lPFC), whole-brain cluster-corrected at *Z* > 2.3, *p* < 0.05. These prefrontal regions spatially overlapped with prefrontal cortex showing a main effect of awareness (this overlap is shown in purple in Fig. 4). In other words, both dmPFC and lPFC regions where activation increased with awareness also coupled inversely with the amygdala to predict a reduction of affective coloring when individuals were aware of the presentation of negative cues.

Critically, in the unaware condition, the association between amygdala-PFC coupling and behavior was not found in the lPFC (ρ = 0.24, *p* = 0.19) or in the dmPFC (ρ = 0.19, *p* = 0.23) (Fig. 3B,C). Further, awareness significantly modulated the association between amygdala-PFC coupling and subsequent evaluative behavior (amygdala-dmPFC 95% CI_slope difference_ = −1.19, −0.35; amygdala-lPFC 95% CI_slope difference_: −1.17, −0.33). (The awareness modulation of the relationship between amygdala-PFC connectivity and affective coloring was also denoted by an unbiased, whole-brain corrected contrast, *Z* > 2.3, *p* < 0.05; Table 3.). These results indicate that in the absence of conscious awareness, this prefrontal circuitry targeting the amygdala fails to modulate behavior.

### Amygdala-PFC white matter microstructure is associated with inverse amygdala-dmPFC functional coupling and less affective coloring specifically following aware processing of negative stimuli

The above-reported amygdala-PFC functional connectivity results underscore the relevance of the amygdala-PFC circuitry for the modulation of emotional processing when a negative cue is available for conscious awareness. The rich inter-individual variability revealed by the functional connectivity analysis when individuals were aware of fearful faces raises the possibility that stable, enduring individual differences in the structural connectivity between the amygdala and PFC may influence both the state of amygdala-PFC coupling and subsequent behavioral outcomes during conscious emotional processing. As mentioned, the amygdala is connected to the PFC primarily via a white matter tract called the uncinate fasciculus. Although connectivity between the amygdala and PFC through the uncinate is bidirectional, higher structural connectivity in the uncinate as reflected by diffusion imaging metrics such as fractional anisotropy (FA) has been typically associated with adaptive emotion-regulatory outcomes in healthy individuals, such as lower anxiety^27^,^33^,^34^. Therefore, we examined whether uncinate FA was associated with inverse amygdala-PFC coupling and reduced affective coloring *specifically* when individuals were aware of being exposed to a negative stimulus. A demonstration of specificity in the relationship between amygdala-PFC white matter microstructure and adaptive outcomes after negative-stimulus processing *to the consciously aware condition* would provide additional evidence that the utility of this circuitry is promoted by conscious awareness. To identify the uncinate fasciculus, a well-described ROI approach in conjunction with deterministic tractography was used (Fig. 5A, cf.^27^,^48^).

Greater FA in the left uncinate fasciculus predicted inverse amygdala-dmPFC functional connectivity (ρ = −0.53, *p* = 0.002) and reduced affective-coloring behavior (ρ = 0.43, *p* = 0.014) when individuals were aware of the emotional stimuli (Fig. 5C,D). In contrast, uncinate FA was unrelated to amygdala-dmPFC connectivity (ρ = 0.1, *p* > 0.5) or to behavior (ρ = 0.09, *p* > 0.6) in the unaware condition—those associations were again specific to the aware condition (uncinate FA and functional connectivity: aware vs. unaware 95% CI_slope difference_ = −0.98, −0.11; uncinate FA and affective coloring: aware vs. unaware 90% CI_slope difference_ = 0.01, 0.66). Right uncinate FA was not as strongly associated with emotional coloring behavior, ρ = 0.33, *p* = 0.065, and not significantly associated with amygdala-dmPFC connectivity, *p* > 0.44.

### Amygdala-dmPFC inverse functional coupling mediates the association between amygdala-PFC white matter microstructure and reduced affective coloring following aware processing of negative stimuli

Given that both functional and structural amygdala-dmPFC connectivity indices were associated with reduced affective coloring specifically when individuals were aware of fearful faces, and that those amygdala-PFC connectivity indices correlated (ρ = −0.53, *p* = 0.002), we tested whether they accounted for shared or independent variance in aware affective coloring behavior using a mediation model. Mediation models require demonstrating that a plausible mediator (amygdala-dmPFC functional connectivity during aware emotional processing) covaries with the outcome variable (affective coloring following aware emotional processing) independently of the independent variable (uncinate FA; cf.^49^). Further, a full mediation model requires the relationship between the independent variable (uncinate FA) and the outcome (affective coloring behavior) to become significantly attenuated (or eliminated) when the mediating variable (amygdala-dmPFC functional connectivity) is included in the same model.

Hence, we entered amygdala-dmPFC functional connectivity weights together with uncinate FA in a simultaneous linear regression model predicting aware affective coloring, and found that inverse amygdala-dmPFC connectivity continued to predict reduced affective coloring even after controlling for the influence of uncinate FA, B = −0.05, *t* = −3.24, *p* = 0.003. Conversely, the relationship between uncinate FA and affective coloring, initially significant, B = −6.17, *t* = −2.80, *p* = 0.009, was attenuated and became non-significant after including amygdala-dmPFC functional connectivity in the model, B = −2.81, *t* = −1.2, *p* > 0.2, suggesting a mediation effect (Fig. 6).

A bootstrapping approach (5,000 bootstrap samples^50^) provided further evidence for a mediation: Amygdala-dmPFC functional connectivity significantly mediated the relationship between uncinate FA and affective coloring, B = −3.35, SE = 1.46, 95% CI = [−1.09, −7.03]. This suggests that more robust amygdala-PFC white matter microstructure may be associated with less affective coloring following conscious emotional processing by promoting inverse amygdala-dmPFC coupling.

### Amygdala-PFC white matter microstructure predicts the state of amygdala-dmPFC functional coupling specifically during aware processing of negative stimuli

Finally, to probe the *valence* specificity in the association between amygdala-PFC white matter microstructure and amygdala-dmPFC functional coupling, we tested a moderation model including valence, awareness, and uncinate FA as factors predicting amygdala-dmPFC functional coupling in a 2 × 2 mixed-model multivariate analysis of covariance (MANCOVA). In this model, Awareness (Aware vs. Unaware) and Valence (Negative: fearful faces vs. Neutral: flowers) were entered as within-subjects factors, and left uncinate FA was entered as a continuous between-subjects variable. A significant 3-way interaction between uncinate FA, valence and awareness (*F*(1,29) = 5.72, *p* = 0.02) indicated that individuals with higher uncinate FA showed more inverse amygdala-dmPFC coupling specifically during exposure to *fearful faces* (i.e., not to flowers) in the aware condition (see Fig. 7 for a depiction of the modulation of amygdala-dmPFC functional coupling by valence and awareness as a function of participants’ uncinate FA quartile). In contrast, individuals with lower uncinate FA showed an *increase* in dmPFC-amygdala functional coupling when fearful faces were visible. Thus, the direction of amygdala-dmPFC coupling during conscious processing of negative cues depended on the robustness of the white matter microstructure supporting the amygdala-PFC circuitry.

## Discussion

The present findings provide a novel framework for understanding how and why conscious processing of emotional information can confer emotion-regulatory benefits. Specifically, these results suggest that conscious awareness of a negative stimulus is required for amygdala-PFC circuitry to modulate subsequent behavior, a finding that was evidenced by both structural and functional connectivity analyses. Furthermore, this study highlights a critical role for individual differences in the circuitry engaged in the affect misattribution paradigm. These individual differences were accounted for by two distinct sets of neural mechanisms: (1) “bottom-up” emotional-stimulus encoding by the amygdala, which was associated with affective coloring behavior only in the *unaware* condition; and (2) “top-down”, potentially regulatory PFC interactions with the amygdala, as reflected by both structural connectivity and functional coupling during negative emotional processing, which explained behavior only in the *aware* condition.

When the negative stimuli (fearful faces) were not available for conscious processing, BOLD signal changes in the amygdala were associated with a subsequent negative bias in evaluative behavior. This is consistent with the idea that the amygdala is a region involved in early appraisal of emotionally salient stimuli^51^,^52^, and that affective misattribution in the absence of awareness correlates with the magnitude of prior affective processing^7^. A number of studies have shown that the amygdala responds to negative facial expressions regardless of visual awareness^3^,^4^,^45^, and that awareness by itself does not necessarily modulate the magnitude of amygdala activation. For example, amygdala engagement in response to fear-relevant stimuli is equivalent in sighted and blind fields of blindsight patient GY^16^, and is also equivalent across subjectively perceived vs. “neglected” fields of patients with hemispatial neglect due to parietal damage^17^. Similarly, in our study, the magnitude of right central amygdala responses to fearful faces was comparable across aware and unaware conditions. Collectively, these findings suggest that subjective awareness of biologically meaningful socio-emotional stimuli is not a pre-requisite for amygdala engagement. Despite equivalent levels of amygdala engagement across awareness conditions, the present results suggest that awareness does modulate the behavioral correlates of amygdala activation, such that only unaware amygdala processing was associated with a negative bias toward subsequently processed stimuli. Thus, our findings extend the behavioral significance of amygdala responses following unaware emotional-stimulus processing beyond the contexts of blindsight and fear conditioning^15^,^53^.

When individuals were aware of the stimuli, the magnitude of amygdala responses to fearful faces did not significantly predict subsequent evaluative behavior. Instead, indices of amygdala-PFC connectivity previously implicated in emotion-regulatory success^26^,^27^,^28^,^31^,^32^,^33^,^34^, including the inverse functional coupling between amygdala-dmPFC and amygdala-lPFC, correlated with less affective coloring. These same indices of amygdala-PFC coupling were *not* associated with affective coloring following unaware emotional-stimulus processing. These findings extend the results of a prior study indicating that aware processing of briefly flashed fearful faces was characterized by more inverse amygdala-PFC coupling^54^, and reinforce the idea that unaware behavioral effects likely reflect a primarily bottom-up emotional-stimulus encoding that is less influenced by interactions with higher-order, putative emotion-regulatory regions (such as PFC). Consistently, the prefrontal regions where coupling with the amygdala predicted less affective coloring in the aware condition spatially overlapped with prefrontal cortex where activation increased during aware (compared to unaware) processing. Collectively, these results suggest that when the source of affect is available for conscious processing, behavior is less susceptible to low-level, emotional-stimulus encoding processes, and may instead incorporate PFC-dependent computations that recruit top-down, modulatory mechanisms.

Accordingly, amygdala-PFC white matter microstructure consistent with higher structural integrity of this circuitry dovetailed with the amygdala-PFC coupling findings, as only in the aware condition did higher uncinate FA predict reduced affective coloring and inverse amygdala-dmPFC coupling. In addition, amygdala-dmPFC functional coupling mediated the association between uncinate FA and affective coloring in the aware condition. Collectively, these findings suggest that, following a consciously processed negative event, a more robust amygdala-prefrontal white matter microstructure may enable the amygdala-prefrontal circuitry to change dynamically in an adaptive manner.

The present results obtained during the passive (aware) viewing of negative stimuli extend the adaptive relevance of both structural and functional markers of amygdala-PFC interactions beyond the contexts of voluntary emotion regulation^26^ and clinical samples^27^,^28^ to an uninstructed, automatic emotion-processing paradigm in healthy individuals. In prior studies, inverse amygdala-PFC coupling had been associated with favorable emotion-regulatory outcomes such as greater success in reappraisal of negative emotion^26^, lower neuroticism^32^ and lower anxiety^28^. Similarly, amygdala-PFC structural connectivity had been negatively associated with trait anxiety^33^, and reported to be lower in individuals diagnosed with generalized anxiety disorder^27^. Our study therefore highlights the functional significance of task-based inverse amygdala-PFC coupling, as well as amygdala-PFC white matter microstructure, beyond the particularities of specific paradigms or clinical status to underscore that they both provide important markers of individual differences in emotional processing—even when the provocative stimuli are rather mild in their intensity (i.e., fearful faces), and when individuals are not instructed to adopt an emotion-regulatory strategy. The fact that this circuitry appears to operate automatically, in response to commonly occurring faces, and that it correlates with social judgments suggests that it likely has important relevance for how ordinary emotional stimuli are processed in daily life.

While the present study of healthy individuals using continuous flash suppression revealed that awareness modulated the behavioral correlates of amygdala activation and amygdala-prefrontal connectivity, prior work conducted in individuals with blindsight and hemispatial neglect has demonstrated that awareness can directly impact the magnitude and time course of peripheral-physiological responses to emotional stimuli, attenuating the magnitude and shortening the time course of those responses^20^,^55^. The neural substrates underlying these various forms of emotion regulation may vary as a function of the specific response system impacted by awareness—e.g., regulation of peripheral-physiological responses likely relies on PFC substrates more directly connected with hypothalamic and brainstem modulators of peripheral-physiological response systems, such as the ventromedial PFC network^56^, in contrast with the dorsomedial and lateral PFC regions highlighted here. Future neuroimaging work examining the output of different emotional response systems measured simultaneously (such as physiological, behavioral, and subjective-experiential) is needed to determine the extent to which specific response systems impacted by awareness—as well as the prefrontal substrates underlying their modulation—generalize across distinct methods of manipulating awareness in healthy and clinical populations.

The following limitations of the present investigation warrant further study. First, the contrast of fearful faces vs. flowers adopted here, while amygdala-engaging, does not isolate whether fearful-face valence or intensity (i.e., arousal) primarily contributed to produce amygdala responses, amygdala-prefrontal interactions and their association with subsequent neutral-face judgments—nor does it address a possible role of stimulus sociality in these effects. The amygdala responds to both stimulus intensity and valence, where its response magnitude likely reflects a combination of both factors^57^. Regarding sociality, extensive previous work has demonstrated affective coloring from social to non-social stimuli (e.g.^8^,^22^,^24^) and vice versa^19^—thus, affective coloring seems to occur independently of sociality. Nonetheless, future work adopting also e.g. happy faces as control stimuli will reveal the specific contributions of emotional-stimulus valence, intensity, and sociality to amygdala reactivity and amygdala-prefrontal interactions.

Second, although the inverse coupling between the amygdala and PFC as revealed by the PPI analysis is consistent with a suppressive association between those two regions, such inverse coupling does not constitute proof of an inhibitory effect. Functional connectivity as estimated in neuroimaging data is inherently correlational— therefore, future work combining TMS/fMRI in humans, and multimodal imaging (including electrophysiology) in non-human primates will be necessary to specify the precise nature and directionality of the inverse amygdala-prefrontal coupling during emotional processing.

In conclusion, we demonstrated that conscious awareness of an emotional stimulus changes the behavioral fate of amygdala responses and amygdala-prefrontal interactions—and that such awareness is particularly beneficial for individuals with greater amygdala-prefrontal structural connectivity. Together, these results pave the way for future studies investigating how therapeutic approaches that rely on conscious awareness of negative events may be tailored to different individuals.

## Materials and Methods

### Participants

We recruited 40 right-handed individuals (21 females; mean age = 20.65, SD = 1.65, range = 18–26) with normal or corrected-to-normal vision from the community in Madison, WI. Individuals were screened and excluded based on standard MRI compatibility criteria, current usage of psychotropic medications, or if they had ever been diagnosed with major depression, anxiety, bipolar disorder, schizophrenia, schizoaffective disorder, or eating disorders. For data analysis, we excluded the data of participants who did not experience robust suppression of stimulus visibility during CFS as evidenced by 2-alternative forced choice (2AFC) stimulus identification performance and subjective reports (for details, see “Stimulus Awareness Assessment” in *Methods).* The data of 31 participants were retained for analysis (19 females; mean age = 20.81, SD = 1.75, range = 18–26). The University of Wisconsin-Madison Health Sciences Institutional Review Board approved all study procedures, which were carried out in accordance with the approved guidelines. All participants provided informed consent and were paid for participation.

### Experimental Design

Data were collected in two sessions: An fMRI session with the Emotion Processing Task; and a 2AFC stimulus detection task outside of the scanner to ensure the effectiveness of stimulus visibility suppression via the CFS manipulation.

### Stimuli

CFS stimuli consisted of 80 Mondrian-patterned images created by drawing rectangles of random colors at random locations in a 3.2° × 3.2° square. Following the paradigm successfully implemented in our prior investigation^7^, we selected affective stimuli consisting of 16 fearful faces (half female) and 16 pictures of flowers subtending 3.2° × 3.2° and matched on average luminance and Root Mean Square contrast.

Fearful faces were selected from the Macbrain Face Stimulus Set (http://www.macbrain.org/resources.htm) and cropped to remove hair and neck. Pictures of flowers were obtained online under the Creative Commons license (http://commons.wikimedia.org/). Scrambled versions of these stimuli (used for the 2AFC stimulus detection task) were created by segmenting the stimuli into square grids, which were randomly rearranged.

To assess affective coloring, 80 neutral faces (half female) were chosen from the XM2VTSDB multi-modal face database project (http://www.ee.surrey.ac.uk/Research/VSSP/xm2vtsdb) and resized to 4.5° × 5.7° rectangles. Note that neither neutral-face identity nor size overlapped with the fearful faces (hence preventing potential priming from lower-order effects).

### Procedure

#### Emotion Processing Task

The experiment consisted of a 2 (Valence: Negative: fearful faces, Neutral: flowers) × 2 (Awareness: Aware, Unaware) within-subjects design. To accentuate affect elicitation, valence and awareness were manipulated in blocks. Every participant was exposed to one set of unaware and one set of aware blocks of each fearful faces and flowers, yielding a total of 4 stimulus-presentation blocks. Stimulus assignment to aware (i.e., stimulus presented to both eyes) vs. unaware (i.e., stimulus presented during CFS) conditions was counterbalanced across participants. Block order was counterbalanced such that both unaware blocks either preceded or followed both aware blocks; whether unaware or aware blocks occurred first, as well as order of valence block (fearful faces vs. flowers) within aware and unaware blocks was randomized and counterbalanced across participants. Each neutral face used to examine affective coloring was presented only once, and randomly assigned to valence and awareness conditions.

**Figure 1:**
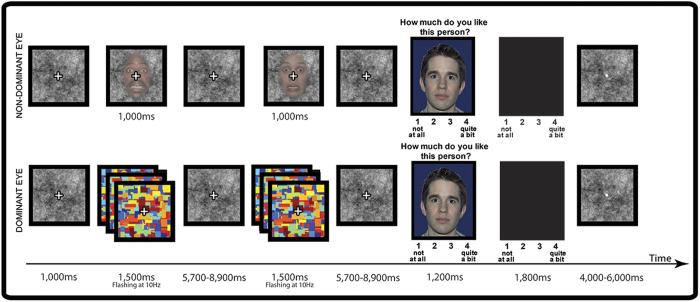
Schematic representation of the experimental trial structure. Negative (fearful faces) and neutral (flowers) stimuli were shown for 1 sec in a blocked fashion. Conscious awareness of these stimuli was manipulated within-subjects. In stimulus-unaware blocks, the presentation of high-contrast, flashing Mondrian patterns to participants’ dominant eye precluded the visibility of stimuli shown to their non-dominant eye. In aware blocks, stimuli were presented to both dominant and non-dominant eyes, and were thus fully visible. Participants were asked to rate the likeability of novel neutral faces using their immediate first impression, thereby providing an index of affective coloring. (Note that this image is not covered by the Creative Commons Attribution license—photographs are from the NimStim Face Stimulus Set. Development of the MacBrain Face Stimulus Set was overseen by Nim Tottenham and supported by the John D. and Catherine T. MacArthur Foundation Research Network on Early Experience and Brain Development. (http://www.macbrain.org/resources.htm).

**Figure 2:**
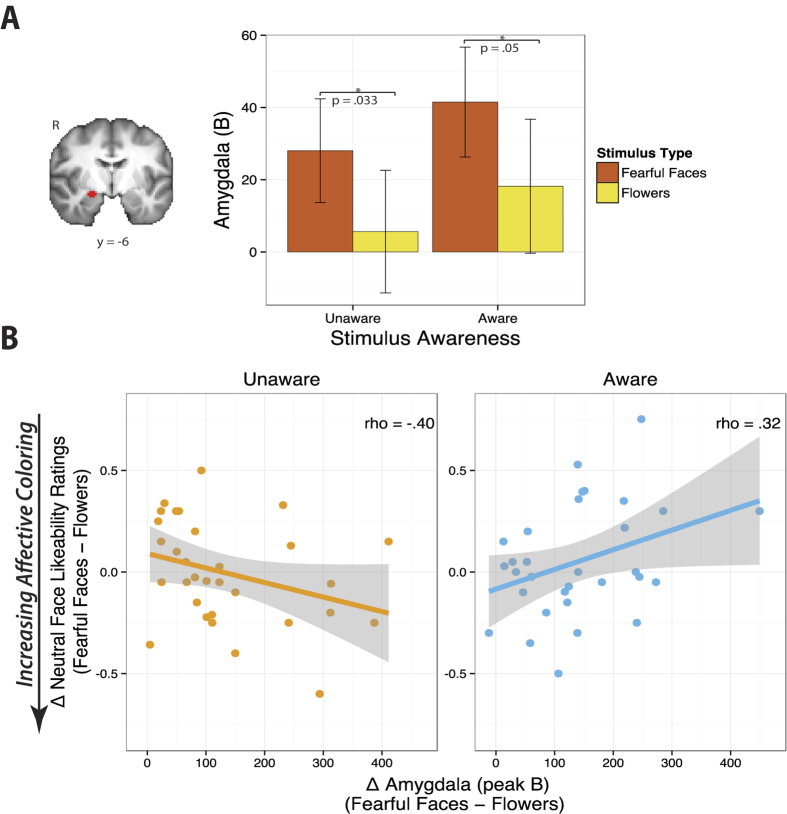
(**A**) Fearful faces increased right central amygdala BOLD response independently of conscious awareness. Using an anatomical ROI approach focused on the central amygdala, we found that right amygdala activation was significantly modulated by valence [fearful faces > flowers] in the unaware, *p* = 0.033, as well as in the aware condition, *p* = 0.05, with no valence by awareness interaction, *p* > 0.8. The right amygdala mask is displayed on the average normalized T1 images across all subjects. Error bars represent the within-subjects 95% confidence intervals per condition^72^. **(B**) Conscious awareness significantly altered the behavioral fate of amygdala responses to fearful faces (relative to flowers). Greater amygdala responses to fearful faces (relative to flowers) were associated with reduced liking of subsequently presented neutral faces, but only when participants were unaware of the emotional stimuli, *p*_*Unaware*_ = 0.026 vs. *p*_*Aware*_ = 0.076; *p*_slope difference_ < 0.01. Here (and on subsequent scatterplots) each data point denotes a subject.

**Figure 3:**
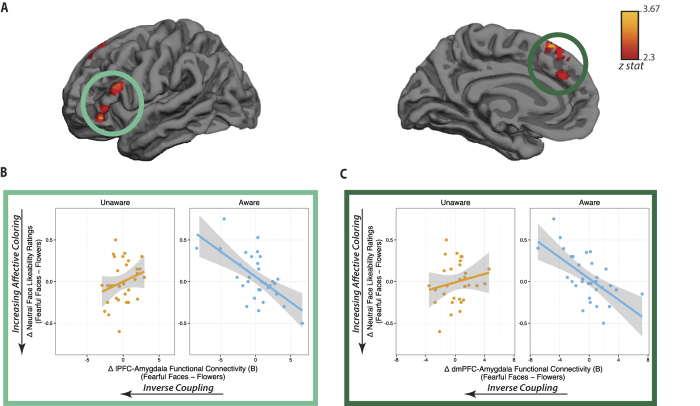
(**A**) During consciously aware processing of fearful faces, reduced negative bias toward novel neutral faces shown seconds later is associated with the inverse coupling between the right amygdala and left lateral and bilateral medial regions of the PFC, whole-brain corrected at *Z* > 2.3, *p* < 0.05. **(B**,**C**) Conscious awareness significantly changed the behavioral correlates of amygdala-PFC coupling, as seen by the significant difference of correlation coefficients when comparing aware vs. unaware associations between affective coloring and amygdala coupling with **(B)** lateral and **(C)** dorsomedial PFC, *p*s_slope difference_ < 0.05. (Note that the scatterplots are depicted here for display purposes only, to illustrate the contrast between aware and unaware conditions. See Table 3 for the results of an unbiased, whole-brain corrected examination of the impact of awareness on these associations).

**Figure 4:**
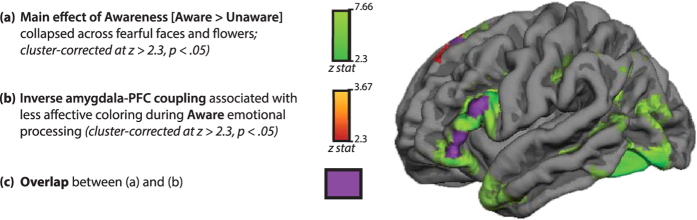
The main effect of awareness (aware – unaware) is shown in green, whole-brain cluster-level corrected for multiple comparisons at *Z* > 2.3, p < 0.05. The result of the regression of affective coloring behavior in the aware condition (i.e., neutral face likeability ratings following [fearful faces – flowers]) on amygdala functional connectivity (following [fearful faces – flowers]) is shown in **red** (i.e., same map as shown on Fig. 3A), and the overlap between these maps is shown in **purple**.

For the unaware blocks, we used CFS^35^: We flashed Mondrian-patterned images flashed to participants’ dominant eye for 1500 ms at 10 Hz, while a static, low-contrast (negative or neutral) stimulus was presented to their non-dominant eye during the first 1000 ms (see Fig. 1). In aware blocks, the low contrast stimuli were presented for 1000 ms to both dominant and non-dominant eyes, and were thus fully visible. Participants were asked to remain still and maintain central fixation throughout the experiment. Prior to unaware blocks, they were told that another image may be presented simultaneously with the colorful squares, and asked to indicate with a button press if they ever thought they saw an image in addition to the squares.

In each block, each of 8 unique stimuli was presented five times. Trials started with a 1000 ms 0.5° × 0.5° fixation cross, followed by the affective stimulus, which was followed by a 5700 ms–8900 ms (7000 ms average) inter-trial interval (ITI). In half the trials, the ITI was followed by a neutral-face likeability rating prompt, where individuals were asked to rate how much they liked a novel neutral face (“How much do you like this person?”) with a button box using a 1–4 scale, where “1” = “not at all”; and “4” = “quite a bit”. Participants were instructed to report on their immediate impression of the faces. Neutral faces were presented for 1200 ms, followed by a blank screen with the response choices for another 1800. Responses were only accepted within the 3000 ms time window. A 4000–6000 ms (5000 ms average) ITI followed.

#### 2AFC Task

Since it is critical that participants experienced robust suppression of stimulus visibility during CFS, in addition to requesting that participants press a button in the event of image breakthrough during the Emotion Processing Task in the scanner, we also examined the effectiveness of CFS for each individual by testing stimulus-identification performance in a 2AFC procedure outside of the scanner (cf.^7^,^45^). To do so, we emulated viewing conditions used in the scanner by employing an adjustable mirror stereoscope mounted on a chin rest to present images displayed on an LCD monitor (60 Hz) at a 50 cm viewing distance in a darkened room. The 2AFC performance was assessed using the same set of 16 stimuli assigned to participants’ unaware blocks (8 fearful faces and 8 flowers). Each unique stimulus was presented 8 times, totaling 128 trials. The 2AFC trials began with a 1000 ms fixation cross, which was followed by 2 successive 1500 ms CFS (1000 ms stimulus)-presentation intervals, interleaved by a 500 ms break. The intact stimulus was randomly presented in the first or second interval, and a scrambled version of the same stimulus was presented in the other interval. At the end of every trial, participants pressed one of 2 buttons to report their best guess as to which interval contained the intact image per the nature of the 2AFC paradigm.

### Image Acquisition

Functional and anatomical data were acquired with a 3.0 T GE scanner (GE Healthcare, Waukesha, WI) using an 8-channel coil. Functional image acquisition used a T2*-weighted gradient-echo, echo-planar imaging (EPI) pulse sequence (40 sagittal slices, 4 mm thickness, 0 mm interslice gap; 64 × 64 matrix, 240 mm field of view (FOV); 2000 ms repetition time (TR); 25 ms echo time (TE); 60° flip angle; 295 image volumes per run). Immediately following acquisition of functional images, high-resolution 3D T1-weighted inversion recovery fast gradient echo anatomical images were collected in 160 contiguous 1.0-mm axial slices (TE = 3.2 ms; TR = 8.2 ms; flip angle = 12°; FOV = 256 × 256 mm; 256 × 256 data acquisition matrix, inversion time TI = 450 ms). Lastly, diffusion tensor imaging was acquired using a spin-echo, single-shot, echo planar imaging (EPI) sequence with diffusion-weighting in 70 non-collinear encoding directions with a diffusion weighting of 1800 s/mm^2^ and six non-diffusion weighted (b = 0) reference images. Sixty-four axial slices were acquired covering the cerebrum (TR = 7500 ms; TE = 72.7 ms; FOV = 230 mm matrix size 100 × 100; 2 mm × 2 mm × 2.3 mm voxels). In order to minimize magnetic field inhomogeneity and EPI distortions, high order shimming was performed and field map images were acquired prior to the DTI acquisition.

### Data Processing and Analysis

Paired samples t-tests, correlations and regression models were run using SPSS version 21.0 (Chicago, IL). The alpha level for all analyses was set to p < 0.05 (two-tailed).

#### Stimulus-Awareness Assessment

We ascertained that observers included in the analysis were unaware of stimuli during CFS blocks by using both subjective reports during the experiment, as well as 2AFC stimulus-identification performance: First, we examined participants’ subjective reports inside of the fMRI scanner, and deemed the data of participants unusable if they reported stimulus breakthrough in greater than 15/40 trials in either fearful-face or flower condition. Seven individuals (out of 40) were excluded from all analyses based on this criterion. Next, each participant’s 2AFC stimulus-identification performance (outside of the scanner) was evaluated on whether it significantly differed from chance (50%) using a binomial test; first combining across both stimulus categories (fearful faces and flowers), as well as within each category separately [as it is known particular stimulus types, such as fearful faces, may have increased proneness to emerge to conscious awareness relative to neutral stimuli; e.g.,^58^]. Because the validity of comparisons made in this experiment rely critically on participants being *unaware* of the stimuli in CFS blocks, the alpha level when contrasting stimulus detection performance against chance was set to (a conservative) two-tailed *p* < 0.1, and participants whose performance exceeded chance at that level were excluded from all reported analyses. Of remaining 33 individuals, 2 performed significantly better than chance at *p* < 0.1 in the 2AFC task when stimuli were collapsed across categories and were also excluded from all analysis. Within each category (fearful faces and flowers), the performance of all remaining individuals did not differ significantly from chance (all *p*s > 0.1). Performance in the 2AFC for the remaining sample (N = 31) did not significantly differ from chance (Total: 50% vs. 50%, *p* > 0.83; fearful faces: 50% vs. 50%, *p* > 0.94; flowers: 50% vs. 50%, *p* > 0.83). Within the remaining sample (N = 31), occasional trials where observers indicated they saw an image in addition to the colorful squares in the scanner were excluded from the analysis of the neuroimaging and the likeability data (0.17% of trials; 0.08% were fearful-face trials, and 0.09% were flower trials).

#### Diffusion Weighted Imaging and Tractography

Image distortions from eddy currents and head motion were compensated using an affine transformation within the FSL (FMRIB Software Library) package (http://fsl.fmrib.ox.ac.uk/fsl). Distortions resulting from magnetic field inhomogeneities were corrected for with the B0 field map and PRELUDE (phase region expanding labeler for unwrapping discrete estimates) and FUGUE (FMRIB’s utility for geometrically unwarping EPIs) within FSL. FSL’s BET (Brain Extraction Tool) was used to isolate the brain tissue. Tensor fitting was performed using CAMINO (http://cmic.cs.ucl.ac.uk/camino/).

A population-specific standard space template was created using all participants’ DTI images with the Diffusion Tensor Imaging Tookit (DTI-TK) (http://www.nitrc.org/projects/dtitk). The unbiased template is constructed so that both the average diffusion features (e.g. FA) and anatomical features (tract size) in the population are accounted for^59^. The individual tensor maps were normalized to the template with rigid, affine, and diffeomorphic alignments and interpolated to 2 × 2 × 2 mm^3^ voxels. DTI-TK was used to calculate FA maps in normalized space.

**Figure 5:**
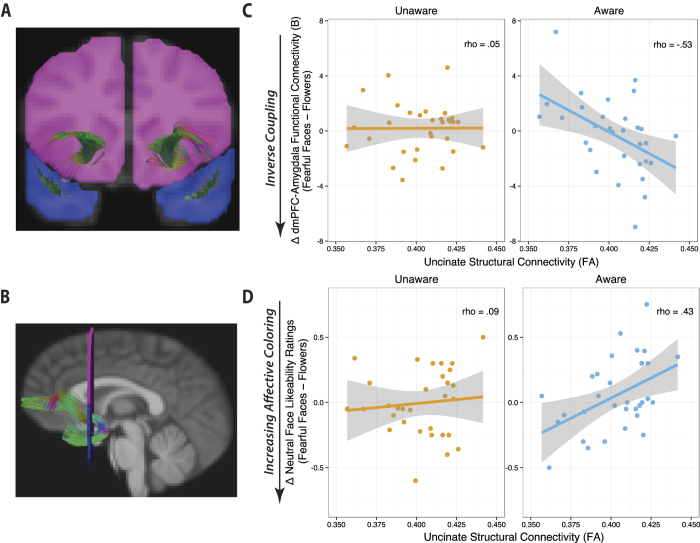
(**A**) Placement of temporal and frontal waypoint regions of interest used to identify the uncinate fasciculus is shown for a representative subject. The Boolean *AND* operation was used to select only the fibers that crossed through both the temporal and frontal waypoint regions of interest. **(B)** The left uncinate fasciculus from a representative subject is shown, overlaid on the average normalized T1 images across all subjects. When individuals were aware of the emotional stimuli [fearful faces – flowers], greater left uncinate fasciculus FA was associated with **(C**) more inverse amygdala-dmPFC coupling, *p* < 0.01 and **(D**) less affective coloring of novel neutral faces, *p* = 0.014.

**Figure 6:**
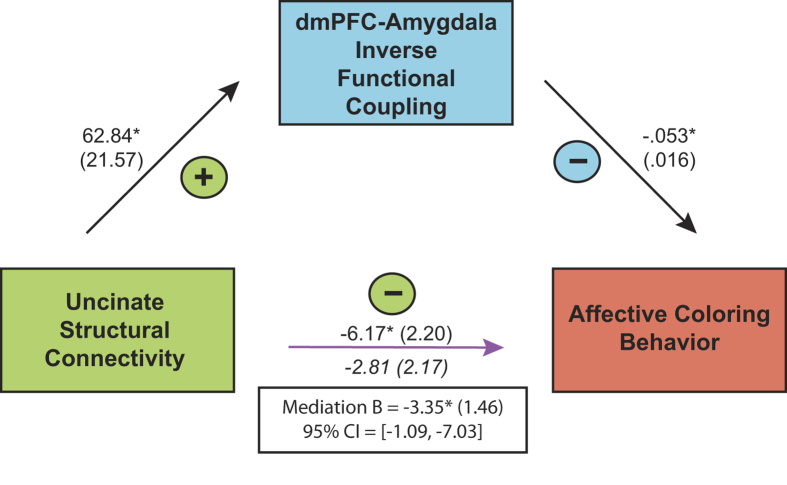
dmPFC-amygdala inverse functional coupling significantly mediated the association between uncinate structural connectivity (FA) and affective coloring behavior following aware processing of negative stimuli [fearful faces – flowers]. Paths are marked with unstandardized coefficients (standard error).

**Figure 7:**
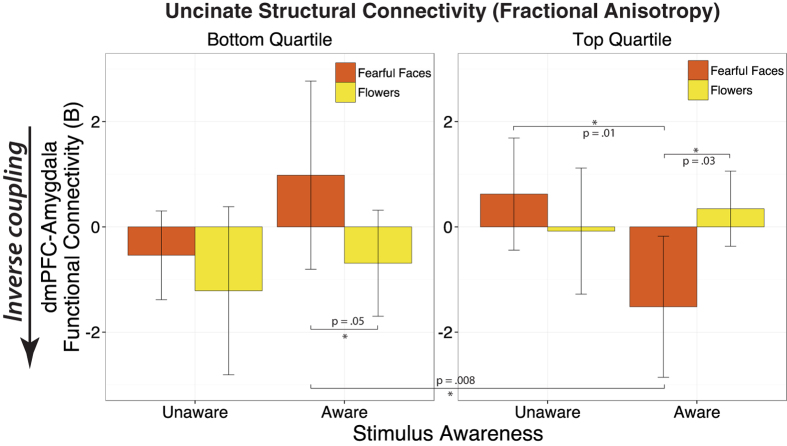
The association between uncinate fasciculus white matter microstructure and the state of dmPFC-amygdala functional coupling is specific to *fearful-face* processing in the *visually aware* condition. Individuals in the top quartile (i.e., with the highest left uncinate fasciculus FA, N = 8) showed a significant *decrease* in dmPFC-amygdala functional coupling when fearful faces were consciously processed (relative to flowers), *p* = 0.03. In contrast, individuals in the bottom quartile (N = 8) of left uncinate FA showed an *increase* in dmPFC-amygdala functional coupling when fearful faces were visible (relative to flowers), *p* = 0.05. This inverse coupling between dmPFC and amygdala during aware fearful-face processing by the high-uncinate group was significantly stronger than when fearful faces were processed non-consciously by those same individuals, *p* = 0.01, and highly significantly stronger than the functional coupling to visible fearful faces shown by the low uncinate group, *p* = 0.008. Error bars represent the within-subjects 95% confidence intervals per condition^72^.

Individual tensor volumes in standard space underwent tractography to reconstruct white matter pathways. The tractography was performed on the whole brain using the TEND algorithm in CAMINO. In order to achieve a seed file for tractography, a white matter mask was defined as FA > 0.15 and the stopping criteria was FA < 0.15. Uncinate fasciculus extraction was performed using a standard protocol^27^,^48^, which entails the placement of temporal and frontal waypoint ROIs in each hemisphere using TrackVis (*AND* conjunction), and averaging FA values for each right and left set of uncinate fasciculus fibers. The frontal and temporal waypoint ROIs used for the uncinate fasciculus extraction are shown in Fig. 5A. We report the results of the original L and R FA variables (which were fully replicated when age and whole-brain FA were controlled for).

#### Functional Neuroimaging

##### Overview

We conducted a region-of-interest analysis to examine whether amygdala encoding of negative stimuli was associated with subsequent affective coloring, and tested whether the association between amygdala encoding and affective coloring differed by visual awareness. Next, upon uncovering that individuals with greater right amygdala responses displayed a negative bias toward unrelated neutral faces in the *unaware* condition only, we conducted whole-brain, voxelwise regressions of affective coloring behavior on right amygdala functional connectivity to examine whether amygdala-prefrontal coupling may have attenuated affective coloring in the aware condition. Following findings revealing that, across individuals, inverse right amygdala-PFC functional coupling was associated with reduced affective coloring specifically in the aware condition, we examined whether individual differences in white matter microstructure of the primary pathway connecting the amygdala to the PFC (the uncinate fasciculus) was also associated with less affective coloring selectively in the aware condition, which would further corroborate the specificity of function of this circuitry to consciously aware processing of emotional stimuli.

#### Functional Neuroimaging Preprocessing & Modeling

Functional neuroimaging data were preprocessed and analyzed using FEAT (FMRIB software library, FSL; www.fmrib.ox.ac.uk/fsl 60). Preprocessing steps included highpass filtering at 100 s, FILM correction for autocorrelation in the BOLD signal, motion correction using MCFLIRT and creation of a confound matrix of points of outlier-intensity changes to be used as regressors of non-interest in the analyses, thus removing movement-confounded activation. Data were smoothed with using a 5 mm full width at half maximum (FWHM) Gaussian spatial filter. Registration of functional images to high-resolution (T1-weighted) structural images and to standard space (MNI152 2 mm template) was carried out in a two-step process using both linear (FLIRT^61^,^62^) and non-linear (FNIRT^63^) algorithms, as follows: First, the registration matrix between the functional EPI images to the high resolution structural image was computed using a linear rigid body (6-DOF) transform. Second, the registration between the high-resolution structural to standard space (i.e. the MNI152 template) was computed using a linear affine (12-DOF) transformation, which was further refined using non-linear (FNIRT) registration at the default 10 mm warp resolution setting. FNIRT uses cubic B-splines functions, bending energy as its regularizing function, and optimizes the sum of squared differences as its cost function. These transformation matrices were then combined and applied to the functional images, and were visually inspected to verify registration quality for each subject.

We used FSL’s 3-level approach to model the data^64^,^65^ (http://fsl.fmrib.ox.ac.uk/fsl/fsl4.0/feat5/detail.html): First, using a canonical hemodynamic response function (γ), separate general linear models were computed for each of the 4 fMRI runs modeling onset times (and their temporal derivative) for the affective stimuli (fearful faces and flowers) and neutral-face rating epochs. Second, a fixed-effects general linear model was computed to combine the parameter estimates of the 4 runs for each participant. Third, when whole-brain associations were examined at the group level, the product of this fixed-effects analysis was the input to a random-effects model (FLAME). Automatic outlier de-weighting was run on a voxelwise basis^66^. Correction for multiple comparisons was performed across the whole-brain by using Gaussian Random Field theory (GRF) at the cluster level at *Z* > 2.3, p < 0.05. All coordinates are reported in MNI space.

#### Functional Neuroimaging Analysis

##### Awareness Main Effect

To verify whether we replicated the previously reported finding of increased prefrontal-cortical engagement during aware compared to unaware stimulus processing^1^ in this sample, aware blocks were contrasted with unaware blocks, i.e., [Aware – Unaware] collapsed across fearful faces and flowers, which we whole-brain cluster-corrected at *Z* > 2.3, p < 0.05.

##### Amygdala Univariate Analysis

To examine whether amygdala encoding of a negative stimulus was involved in subsequent affective coloring behavior, we extracted amygdala activation (during emotional-stimulus exposure [fearful faces – flowers]) from each participant using an amygdalar mask comprising the central nucleus of the amygdala (CeA; Figure S4 in^43^,^44^). The CeA mask was chosen given the well-described role of this amygdalar region as a primary recipient of stimulus-value computations in basal and lateral amygdalar nuclei, and as a major amygdala output center projecting to autonomic nervous system and relevant stimulus-encoding regions of the brain^18^,^67^,^68^.

As previously described, the CeA amygdala ROI prescription was derived from the Mai atlas (see text and Figure S4 on^43^ for details;^44^,^69^). Visual inspection indicated that this approach enhanced anatomical sensitivity and selectivity when compared to the probabilistic “centromedial” amygdala atlas distributed with FSL^70^. The CeA ROI began 4 mm caudal to the rostral margin of the amygdala and continued in the caudal direction for 8 mm. The rostral portion of the ROI was prescribed ventral and medial to the lateral extension of the anterior commissure (AC; i.e., where the AC converges with the uncinate fasciculus). Throughout, the ROI was prescribed lateral to the optic tract and dorsal to the temporal horn of the lateral ventricle. The CeA seed was generated by spatially smoothing (2 mm-voxel dilation, followed by 1-voxel erosion) and decimating (2-mm) the ROI. Using the spatially-normalized T1, we manually verified that the seed was centered within the provisional location of the CeA for each participant.

Using this CeA amygdalar mask here and throughout this report, we extracted the mean amygdalar activation to examine whether emotional stimulus exposure [fearful faces – flowers] increased amygdala activation on average across participants in each aware and unaware condition. Next, we examined if the magnitude of amygdala engagement to the emotional stimuli was associated with affective coloring by correlating the peak amygdala responding for each individual during emotional-stimulus exposure [fearful faces – flowers] with the magnitude of subsequent affective coloring (neutral face likeability ratings following [fearful faces – flowers]) across individuals in each aware and unaware condition. We used a Spearman’s rank coefficient to test these associations given the increased robustness of this method to outliers (found in the aware condition). Next, to examine whether emotional-stimulus encoding by the amygdala was associated with affective coloring differently depending on stimulus awareness, we tested the difference between correlation coefficients obtained in aware and unaware conditions (i.e., aware − unaware) using Zou’s^46^ method, which yields a confidence interval (CI) for the difference of dependent correlation coefficients (where statistical significance at e.g., p < 0.05 is indicated by the 95% CI not including zero)^46^.

##### Amygdala Functional Connectivity

Because emotional-stimulus encoding by the right amygdala was only associated with affective coloring in the unaware condition, and in light of our question regarding whether amygdala-PFC circuitry may be differentially involved in emotional processing depending on awareness, we examined PFC-dependent interactions with the right amygdala. (Univariate and connectivity results for the left amygdala are reported in the Supplementary Information.) Given prior findings relating inverse amygdala-PFC coupling to more favorable emotion-regulatory outcomes^26^,^27^,^28^,^31^,^32^,^71^, we examined whether amygdala-PFC coupling was associated with affective coloring behavior in this emotion misattribution paradigm. To that end, we conducted a psychophysiological interaction analysis (PPI)^47^, also known as task-dependent connectivity, with the right CeA amygdala as a seed. First, we extracted the mean time series data for the right amygdala. Next, we ran a new first-level general linear model including the (demeaned) right amygdala timecourse data as a regressor, and setting up an interaction regressor between the right amygdala time course data and the stimulus (fearful face or flower) regressor for each of the aware and unaware blocks. The result of this interaction regressor is the task-dependent connectivity of the amygdala with each voxel of the brain.

As before, we followed FSL’s 3-level analysis approach. An intermediary fixed-effects analytical step combined connectivity data across blocks for each participant and enabled contrasts between conditions (fearful faces vs. flowers for each awareness condition). At the group level, we examined whether affective coloring was associated with the strength of amygdala-PFC coupling during emotional-stimulus processing by running voxelwise regressions of affective coloring behavior [fearful faces – flowers neutral face likeability ratings] on individuals’ amygdala connectivity contrast maps in each aware and unaware condition [fearful faces – flowers]; (all analyses were cluster-corrected for multiple comparisons at the whole-brain level at *Z* > 2.3, *p* < 0.05). Following a significant association between inverse amygdala-dmPFC and amygdala-lPFC coupling and reduced affective-coloring behavior in the consciously aware condition only, we tested for the specificity of this neural mechanism to the aware condition in 2 ways. First, we extracted the amygdala connectivity weights with the dmPFC and lPFC clusters [fearful faces – flowers], and correlated them with later affective-coloring behavior ([fearful faces – flowers] neutral face likeability ratings) separately for aware and unaware conditions. To test the difference between these correlation coefficients [aware – unaware] we used Zou’s method, as described earlier^46^. As a secondary (and potentially less biased) test of the differential and specific involvement of amygdala-prefrontal connectivity in behavior in the aware condition, we subtracted the maps comprising the correlation between amygdala connectivity and affective coloring while individuals were aware vs. when they were unaware of the emotional stimuli, i.e., (Aware [fearful faces – flowers] correlation between amygdala connectivity and [fearful faces – flowers] neutral-face likeability ratings) – (Unaware [fearful faces – flowers] correlation between amygdala connectivity and [fearful faces – flowers] neutral-face likeability ratings), which we cluster-corrected for multiple comparisons across the whole brain at *Z* > 2.3, *p* < 0.05; Table 3). Both methods converged in highlighting the specificity of amygdala-dmPFC/lPFC functional coupling and affective coloring to the consciously aware condition.

## Additional Information

**How to cite this article**: Lapate, R. C. *et al.* Awareness of Emotional Stimuli Determines the Behavioral Consequences of Amygdala Activation and Amygdala-Prefrontal Connectivity. *Sci. Rep.* **6**, 25826; doi: 10.1038/srep25826 (2016).

## Supplementary Material

Supplementary Information

## Figures and Tables

**Table 1:** MNI coordinates of the areas where activation was significantly greater during aware compared to unaware processing (i.e., [Aware – Unaware] collapsed over fearful faces and flowers); whole-brain cluster-level corrected for multiple comparisons at *Z* > 2.3, *p* < 0.05.

Region	Side	Size (mm^3^)	Coordinates at Z peak			Z peak
			x	y	z	
Lateral Prefrontal Cortex (BA 9/46/45)	L	31224	−50	14	28	5.07
			−48	18	18	4.54
			−48	22	12	4.49
			−48	34	−6	4.46
Dorsomedial Prefrontal Cortex (BA 6/8)	R/L	5976	−6	20	50	4.05
			−4	34	38	3.73
Extrastriate Cortex (fusiform cortex and lateral occipital)	R	63504	44	−68	14	7.41
			44	−54	24	7.29
			56	−50	10	5.47
Extrastriate Cortex (fusiform cortex and lateral occipital)	L	50680	−42	−70	14	7.66
			−44	−54	16	7.03

**Table 2:** MNI coordinates of the PFC clusters (and their local maxima) whose inverse functional coupling with the right amygdala during aware fearful-face viewing (relative to flowers) predicted reduced affective coloring as indexed by higher likeability ratings of novel neutral faces following the presentation of [Fearful Faces – Flowers], whole-brain cluster-level corrected for multiple comparisons at *Z* > 2.3, *p* < 0.05.

Region	Side	Size (mm^3^)	Coordinates at Z peak			Z peak
			x	y	z	
Lateral Prefrontal Cortex Middle/Inferior Frontal Gyrus (BA 9/46/45)	L	4104	−56	16	24	3.36
			−52	22	20	3.33
			−48	36	−6	3.27
			−48	32	−6	3.22
			−48	18	6	3.06
			−48	26	2	2.79
Dorsomedial Prefrontal Cortex (BA6/8)	R/L	3832	−4	26	54	3.67
			4	40	42	3.52

**Table 3:** MNI coordinates of the areas (and their local maxima) whose inverse functional coupling with the right amygdala during emotional processing [Fearful faces – Flowers] was associated with reduced affective coloring (as indexed by higher likeability ratings of novel neutral faces following the presentation of [Fearful faces – Flowers]) *differently* depending on visual awareness (Aware – Unaware), whole-brain cluster-level corrected for multiple comparisons at *Z* > 2.3, *p* < 0.05.

Region	Side	Size (mm^3^)	Coordinates at Z peak			Z peak
			x	y	z	
Lateral Prefrontal Cortex (BA 9/46/45)	L	30464	−48	28	18	4.81
Dorsomedial Prefrontal Cortex (BA 6/8)	R/L	17360	−4	30	54	4.61
Inferior Parietal/Angular Gyrus	R/L	4368	−56	−60	32	3.86
Posterior Insula	R/L	3528	−42	−30	10	3.88

## References

[b1] Dehaene S. & Changeux J.-P. Experimental and theoretical approaches to conscious processing. Neuron 70, 200–27 (2011). 21521609 10.1016/j.neuron.2011.03.018

[b2] Lau H. C. & Rosenthal D. Empirical support for higher-order theories of conscious awareness. Trends Cogn. Sci. 15, 365–73 (2011). 21737339 10.1016/j.tics.2011.05.009

[b3] Williams M. A., Morris A. P., McGlone F., Abbott D. F. & Mattingley J. B. Amygdala responses to fearful and happy facial expressions under conditions of binocular suppression. J. Neurosci. 24, 2898–904 (2004). 15044528 10.1523/JNEUROSCI.4977-03.2004 PMC6729857

[b4] Whalen P. J. *et al.* Masked presentations of emotional facial expressions modulate amygdala activity without explicit knowledge. J. Neurosci. 18, 411–8 (1998). 9412517 10.1523/JNEUROSCI.18-01-00411.1998 PMC6793390

[b5] Olsson A. & Phelps E. A. Learned Fear of ‘Unseen’ Faces after Pavlovian, Observational, and Instructed Fear. Psychol. Sci. 15, 822–828 (2004). 15563327 10.1111/j.0956-7976.2004.00762.x

[b6] Raio C. M., Carmel D., Carrasco M. & Phelps E. A. Nonconscious fear is quickly acquired but swiftly forgotten. Curr. Biol. 22, R477–9 (2012). 22720676 10.1016/j.cub.2012.04.023 PMC3758872

[b7] Lapate R. C., Rokers B., Li T. & Davidson R. J. Nonconscious emotional activation colors first impressions: A regulatory role for conscious awareness. Psychol. Sci. 25, 349–357 (2014). 24317420 10.1177/0956797613503175 PMC4070508

[b8] Almeida J., Pajtas P. E., Mahon B. Z., Nakayama K. & Caramazza A. Affect of the unconscious: visually suppressed angry faces modulate our decisions. Cogn. Affect. Behav. Neurosci. 13, 94–101 (2013). 23224765 10.3758/s13415-012-0133-7 PMC4752568

[b9] Anderson E., Siegel E., White D. & Barrett L. F. Out of sight but not out of mind: unseen affective faces influence evaluations and social impressions. Emotion 12, 1210–21 (2012). 22506501 10.1037/a0027514 PMC4957816

[b10] Freud S. The Ego and the Id (The Standard Edition of the Complete Psychological Works of Sigmund Freud). The Standa, (W. W. Norton & Company, 1990).

[b11] Kabat-Zinn J. *et al.* Effectiveness of a meditation-based stress reduction program in the treatment of anxiety disorders. Am. J. Psychiatry 149, 936–943 (1992). 1609875 10.1176/ajp.149.7.936

[b12] Beck A. T. The evolution of the cognitive model of depression and its neurobiological correlates. Am. J. Psychiatry 165, 969–977 (2008). 18628348 10.1176/appi.ajp.2008.08050721

[b13] Pasley B. N., Mayes L. C. & Schultz R. T. Subcortical discrimination of unperceived objects during binocular rivalry. Neuron 42, 163–72 (2004). 15066273 10.1016/s0896-6273(04)00155-2

[b14] Williams L. M. *et al.* Amygdala-prefrontal dissociation of subliminal and supraliminal fear. Hum. Brain Mapp. 27, 652–61 (2006). 16281289 10.1002/hbm.20208 PMC6871444

[b15] Pegna A. J., Khateb A., Lazeyras F. & Seghier M. L. Discriminating emotional faces without primary visual cortices involves the right amygdala. Nat. Neurosci. 8, 24–5 (2005). 15592466 10.1038/nn1364

[b16] Morris J. S., DeGelder B., Weiskrantz L. & Dolan R. J. Differential extrageniculostriate and amygdala responses to presentation of emotional faces in a cortically blind field. Brain 124, 1241–52 (2001). 11353739 10.1093/brain/124.6.1241

[b17] Vuilleumier P. *et al.* Neural response to emotional faces with and without awareness: event-related fMRI in a parietal patient with visual extinction and spatial neglect. Neuropsychologia 40, 2156–66 (2002). 12208011 10.1016/s0028-3932(02)00045-3

[b18] LeDoux J. E. Rethinking the emotional brain. Neuron 73, 653–76 (2012). 22365542 10.1016/j.neuron.2012.02.004 PMC3625946

[b19] Li W., Moallem I., Paller K. A. & Gottfried J. A. Subliminal smells can guide social preferences. Psychol. Sci. 18, 1044–9 (2007). 18031410 10.1111/j.1467-9280.2007.02023.x

[b20] Tamietto M. *et al.* Unseen facial and bodily expressions trigger fast emotional reactions. Proc. Natl. Acad. Sci. USA 106, 17661–17666 (2009). 19805044 10.1073/pnas.0908994106 PMC2764895

[b21] Schwarz N. & Clore G. L. Mood, Misattribution, and Judgments of Weil-Being : Informative and Directive Functions of Affective States. J. Pers. Soc. Psychol. 45, 513–523 (1983).

[b22] Murphy S. T. & Zajonc R. B. Affect, cognition, and awareness: affective priming with optimal and suboptimal stimulus exposures. J. Pers. Soc. Psychol. 64, 723–739 (1993). 8505704 10.1037//0022-3514.64.5.723

[b23] Sweeny T. D., Grabowecky M., Suzuki S. & Paller K. A. Long-lasting effects of subliminal affective priming from facial expressions. Conscious. Cogn. 18, 929–938 (2009). 19695907 10.1016/j.concog.2009.07.011 PMC2784103

[b24] Rotteveel M., de Groot P., Geutskens A. & Phaf R. H. Stronger suboptimal than optimal affective priming? Emotion 1, 348–364 (2001). 12901397 10.1037/1528-3542.1.4.348

[b25] Brooks S. J. *et al.* Exposure to subliminal arousing stimuli induces robust activation in the amygdala, hippocampus, anterior cingulate, insular cortex and primary visual cortex: a systematic meta-analysis of fMRI studies. Neuroimage 59, 2962–73 (2012). 22001789 10.1016/j.neuroimage.2011.09.077

[b26] Lee H., Heller A. S., van Reekum C. M., Nelson B. & Davidson R. J. Amygdala-prefrontal coupling underlies individual differences in emotion regulation. Neuroimage 62, 1575–1581 (2012). 22634856 10.1016/j.neuroimage.2012.05.044 PMC3408571

[b27] Tromp D. P. M. *et al.* Reduced structural connectivity of a major frontolimbic pathway in generalized anxiety disorder. Arch. Gen. Psychiatry 69, 925–34 (2012). 22945621 10.1001/archgenpsychiatry.2011.2178 PMC3566704

[b28] Etkin A., Prater K. E., Hoeft F., Menon V. & Schatzberg A. F. Failure of anterior cingulate activation and connectivity with the amygdala during implicit regulation of emotional processing in generalized anxiety disorder. Am. J. Psychiatry 167, 545–54 (2010). 20123913 10.1176/appi.ajp.2009.09070931 PMC4367202

[b29] Buhle J. T. *et al.* Cognitive reappraisal of emotion: a meta-analysis of human neuroimaging studies. Cereb. Cortex 24, 2981–90 (2014). 23765157 10.1093/cercor/bht154 PMC4193464

[b30] Urry H. L. *et al.* Amygdala and ventromedial prefrontal cortex are inversely coupled during regulation of negative affect and predict the diurnal pattern of cortisol secretion among older adults. J. Neurosci. 26, 4415–25 (2006). 16624961 10.1523/JNEUROSCI.3215-05.2006 PMC6673990

[b31] Etkin A., Egner T., Peraza D. M., Kandel E. R. & Hirsch J. Resolving emotional conflict: a role for the rostral anterior cingulate cortex in modulating activity in the amygdala. Neuron 51, 871–82 (2006). 16982430 10.1016/j.neuron.2006.07.029

[b32] Cremers H. R. *et al.* Neuroticism modulates amygdala-prefrontal connectivity in response to negative emotional facial expressions. Neuroimage 49, 963–70 (2010). 19683585 10.1016/j.neuroimage.2009.08.023

[b33] Kim M. J. & Whalen P. J. The structural integrity of an amygdala-prefrontal pathway predicts trait anxiety. J. Neurosci. 29, 11614–8 (2009). 19759308 10.1523/JNEUROSCI.2335-09.2009 PMC2791525

[b34] Westlye L. T., Bjørnebekk A., Grydeland H., Fjell A. M. & Walhovd K. B. Linking an anxiety-related personality trait to brain white matter microstructure: Diffusion tensor imaging and harm avoidance. Arch. Gen. Psychiatry 68, 369–377 (2011). 21464361 10.1001/archgenpsychiatry.2011.24

[b35] Tsuchiya N. & Koch C. Continuous flash suppression reduces negative afterimages. Nat. Neurosci. 8, 1096–1101 (2005). 15995700 10.1038/nn1500

[b36] Hariri A. R. *et al.* Serotonin transporter genetic variation and the response of the human amygdala. Science 297, 400–3 (2002). 12130784 10.1126/science.1071829

[b37] Fakra E. *et al.* Effects of HTR1A C(-1019)G on amygdala reactivity and trait anxiety. Arch. Gen. Psychiatry 66, 33–40 (2009). 19124686 10.1001/archpsyc.66.1.33 PMC2736132

[b38] Nikolova Y. S. *et al.* Beyond genotype: serotonin transporter epigenetic modification predicts human brain function. Nat. Neurosci. 17, 1153–5 (2014). 25086606 10.1038/nn.3778 PMC4146649

[b39] Ohman A. & Soares J. J. ‘Unconscious anxiety’: phobic responses to masked stimuli. J. Abnorm. Psychol. 103, 231–240 (1994). 8040492 10.1037//0021-843x.103.2.231

[b40] Somerville L. H., Kim H., Johnstone T., Alexander A. L. & Whalen P. J. Human amygdala responses during presentation of happy and neutral faces: Correlations with state anxiety. Biol. Psychiatry 55, 897–903 (2004). 15110733 10.1016/j.biopsych.2004.01.007

[b41] Cooney R. E., Atlas L. Y., Joormann J., Eugène F. & Gotlib I. H. Amygdala activation in the processing of neutral faces in social anxiety disorder: is neutral really neutral? Psychiatry Res. 148, 55–9 (2006). 17030117 10.1016/j.pscychresns.2006.05.003

[b42] Dannlowski U. & Suslow T. Test-retest reliability of subliminal facial affective priming. Psychol. Rep. 98, 153–8 (2006). 16673968 10.2466/pr0.98.1.153-158

[b43] Birn R. M. *et al.* Evolutionarily conserved prefrontal-amygdalar dysfunction in early-life anxiety. Mol. Psychiatry 19, 915–22 (2014). 24863147 10.1038/mp.2014.46 PMC4111803

[b44] Oler J. A. *et al.* Evidence for coordinated functional activity within the extended amygdala of non-human and human primates. Neuroimage, 61(4), 1059–1066. 22465841 10.1016/j.neuroimage.2012.03.045 PMC3376204

[b45] Jiang Y. & He S. Cortical responses to invisible faces: dissociating subsystems for facial-information processing. Curr. Biol. 16, 2023–9 (2006). 17055981 10.1016/j.cub.2006.08.084

[b46] Zou G. Y. Toward using confidence intervals to compare correlations. Psychol. Methods 12, 399–413 (2007). 18179351 10.1037/1082-989X.12.4.399

[b47] O’Reilly J. X., Woolrich M. W., Behrens T. E. J., Smith S. M. & Johansen-Berg H. Tools of the trade: psychophysiological interactions and functional connectivity. Soc. Cogn. Affect. Neurosci. 7, 604–9 (2012). 22569188 10.1093/scan/nss055 PMC3375893

[b48] Mori S. *et al.* Imaging cortical association tracts in the human brain using diffusion-tensor-based axonal tracking. Magn. Reson. Med. 47, 215–23 (2002). 11810663 10.1002/mrm.10074

[b49] Baron R. M. & Kenny D. A. The moderator-mediator variable distinction in social psychological research: conceptual, strategic, and statistical considerations. J. Pers. Soc. Psychol. 51, 1173–1182 (1986). 3806354 10.1037//0022-3514.51.6.1173

[b50] Preacher K. J. & Hayes A. F. Asymptotic and resampling strategies for assessing and comparing indirect effects in multiple mediator models. Behav. Res. Methods 40, 879–91 (2008). 18697684 10.3758/brm.40.3.879

[b51] Adolphs R. What does the amygdala contribute to social cognition? Ann. N. Y. Acad. Sci. 1191, 42–61 (2010). 20392275 10.1111/j.1749-6632.2010.05445.x PMC2871162

[b52] Pessoa L. Emotion and cognition and the amygdala: from ‘what is it?’ to ‘what’s to be done?’. Neuropsychologia 48, 3416–29 (2010). 20619280 10.1016/j.neuropsychologia.2010.06.038 PMC2949460

[b53] Morris J. S., Ohman A. & Dolan R. J. Conscious and unconscious emotional learning in the human amygdala. Nature 393, 467–70 (1998). 9624001 10.1038/30976

[b54] Williams L. M. *et al.* Mode of functional connectivity in amygdala pathways dissociates level of awareness for signals of fear. J. Neurosci. 26, 9264–71 (2006). 16957082 10.1523/JNEUROSCI.1016-06.2006 PMC6674508

[b55] Tamietto M. *et al.* Once you feel it, you see it: Insula and sensory-motor contribution to visual awareness for fearful bodies in parietal neglect. Cortex 62, 56–72 (2015). 25465122 10.1016/j.cortex.2014.10.009

[b56] Öngür D. & Price J. L. The Organization of Networks within the Orbital and Medial Prefrontal Cortex of Rats, Monkeys and Humans. Cereb. Cortex 10, 206–219 (2000). 10731217 10.1093/cercor/10.3.206

[b57] Winston J. S., Gottfried J. A., Kilner J. M. & Dolan R. J. Integrated neural representations of odor intensity and affective valence in human amygdala. J. Neurosci. 25, 8903–8907 (2005). 16192380 10.1523/JNEUROSCI.1569-05.2005 PMC6725588

[b58] Yang E., Zald D. H. & Blake R. Fearful expressions gain preferential access to awareness during continuous flash suppression. Emotion 7, 882–886 (2007). 18039058 10.1037/1528-3542.7.4.882 PMC4038625

[b59] Zhang H., Yushkevich P. a, Rueckert D. & Gee J. C. Unbiased white matter atlas construction using diffusion tensor images. Med. Image Comput. Comput. Assist. Interv. 10, 211–8 (2007). 18044571 10.1007/978-3-540-75759-7_26

[b60] Smith S. M. *et al.* Advances in functional and structural MR image analysis and implementation as FSL. Neuroimage 23 Suppl 1, S208–19 (2004). 15501092 10.1016/j.neuroimage.2004.07.051

[b61] Jenkinson M. & Smith S. A global optimisation method for robust affine registration of brain images. Med. Image Anal. 5, 143–56 (2001). 11516708 10.1016/s1361-8415(01)00036-6

[b62] Jenkinson M., Bannister P., Brady M. & Smith S. Improved optimization for the robust and accurate linear registration and motion correction of brain images. Neuroimage 17, 825–841 (2002). 12377157 10.1016/s1053-8119(02)91132-8

[b63] Andersson J. L. R., Jenkinson M. & Smith S. Non-linear registration aka Spatial normalisation. *Technical report*. (2007). Available at: https://www.fmrib.ox.ac.uk/analysis/techrep/tr07ja2/tr07ja2.pdf (Accessed: 22nd March 2016).

[b64] Woolrich M. W. *et al.* Bayesian analysis of neuroimaging data in FSL. Neuroimage 45, S173–86 (2009). 19059349 10.1016/j.neuroimage.2008.10.055

[b65] Jenkinson M., Beckmann C. F., Behrens T. E. J., Woolrich M. W. & Smith S. M. FSL. Neuroimage 62, 782–90 (2012). 21979382 10.1016/j.neuroimage.2011.09.015

[b66] Woolrich M. Robust group analysis using outlier inference. Neuroimage 41, 286–301 (2008). 18407525 10.1016/j.neuroimage.2008.02.042

[b67] Price J. & Amaral D. An autoradiographic study of the projections of the central nucleus of the monkey amygdala. J. Neurosci. 1, 1242–1259 (1981). 6171630 10.1523/JNEUROSCI.01-11-01242.1981 PMC6564217

[b68] Adolphs R. & Spezio M. Role of the amygdala in processing visual social stimuli. Prog. Brain Res. 156, 363–78 (2006). 17015091 10.1016/S0079-6123(06)56020-0

[b69] Mai J. K., Assheuer J. & Paxinos G. Atlas of the Human Brain. (Elsevier Academic Press, 2003).

[b70] Amunts K. *et al.* Cytoarchitectonic mapping of the human amygdala, hippocampal region and entorhinal cortex: intersubject variability and probability maps. Anat. Embryol. (Berl). 210, 343–352 (2005). 16208455 10.1007/s00429-005-0025-5

[b71] Mobbs D. *et al.* From threat to fear: the neural organization of defensive fear systems in humans. J. Neurosci. 29, 12236–43 (2009). 19793982 10.1523/JNEUROSCI.2378-09.2009 PMC2782300

[b72] Morey R. Confidence intervals from normalized data: A correction to Cousineau (2005). Tutor. Quant. Methods Psychol. 4, 61–64 (2008).

