# Supplementary material for: Awareness of Emotional Stimuli Determines the Behavioral Consequences of Amygdala Activation and Amygdala-Prefrontal Connectivity
Source: Sci Rep. 2016 May 16;6:25826. doi: 10.1038/srep25826 (PMC4867584; doi:10.1038/srep25826)

Supplementary Information

Awareness of Emotional Stimuli Determines the Behavioral Consequences of Amygdala Activation and Amygdala-Prefrontal Connectivity

R. C. Lapate, B. Rokers, D. P. M. Tromp, N. S. Orfali, J. A. Oler,
S.T. Doran, N. Adluru, A. L. Alexander, R. J. Davidson

Supplementary Results

The analysis of amygdala-prefrontal functional connectivity in the main report focused on the *right* amygdala because the right amygdala significantly responded to fearful faces in both unaware and aware conditions (p = .033 and p = .05, respectively); and yet, only unaware fearful-face processing by the right amygdala was associated with a subsequent negative bias toward novel neutral faces, Spearman's $\rho = -.40$, $p = .026$ (compared to $\rho = .32$, $p = .076$ for the aware condition). In contrast, in the unaware condition, left amygdala activation to fearful faces did not reach statistical significance, $p = .08$, and the correlation between left amygdala activation and subsequent likeability behavior correlation was non significant, $\rho = -.23$, $p = .2$. Following an anonymous reviewer's suggestion, we explored whether in the *consciously aware* condition the functional connectivity of the *left* amygdala would be associated with attenuated affective coloring behavior (as the right amygdala functional connectivity was). While the voxelwise regression of likeability ratings on left amygdala functional connectivity did not reach statistical threshold for multiple comparisons correction at the whole-brain level, PPI weights between the left amygdala and LPFC and dmPFC clusters identified in the right amygdala PPI analysis were reliably associated with less affective coloring in the aware condition, $\rho = -.53$, $p < .001$ and $\rho = -.48$, $p < .001$, respectively (**Supplementary Figure 1A & 1B**). Further, greater left uncinate FA was associated with a more inverse coupling between the left amygdala and dmPFC in response to consciously processed fearful faces, $\rho = -.37$, $p = .036$ (**Supplementary Figure 2**). Collectively, these findings suggest that both right and left amygdalae may be targets of PFC interactions associated with a reduction of emotional-stimulus congruent bias following consciously aware emotional processing.

Supplementary Figure 1. During consciously aware processing of fearful faces, the inverse functional coupling between the left amygdala and **(A)** left lateral PFC and **(B)** dorsomedial PFC was associated with a reduced negative bias toward novel neutral faces shown seconds later (i.e., less affective coloring).

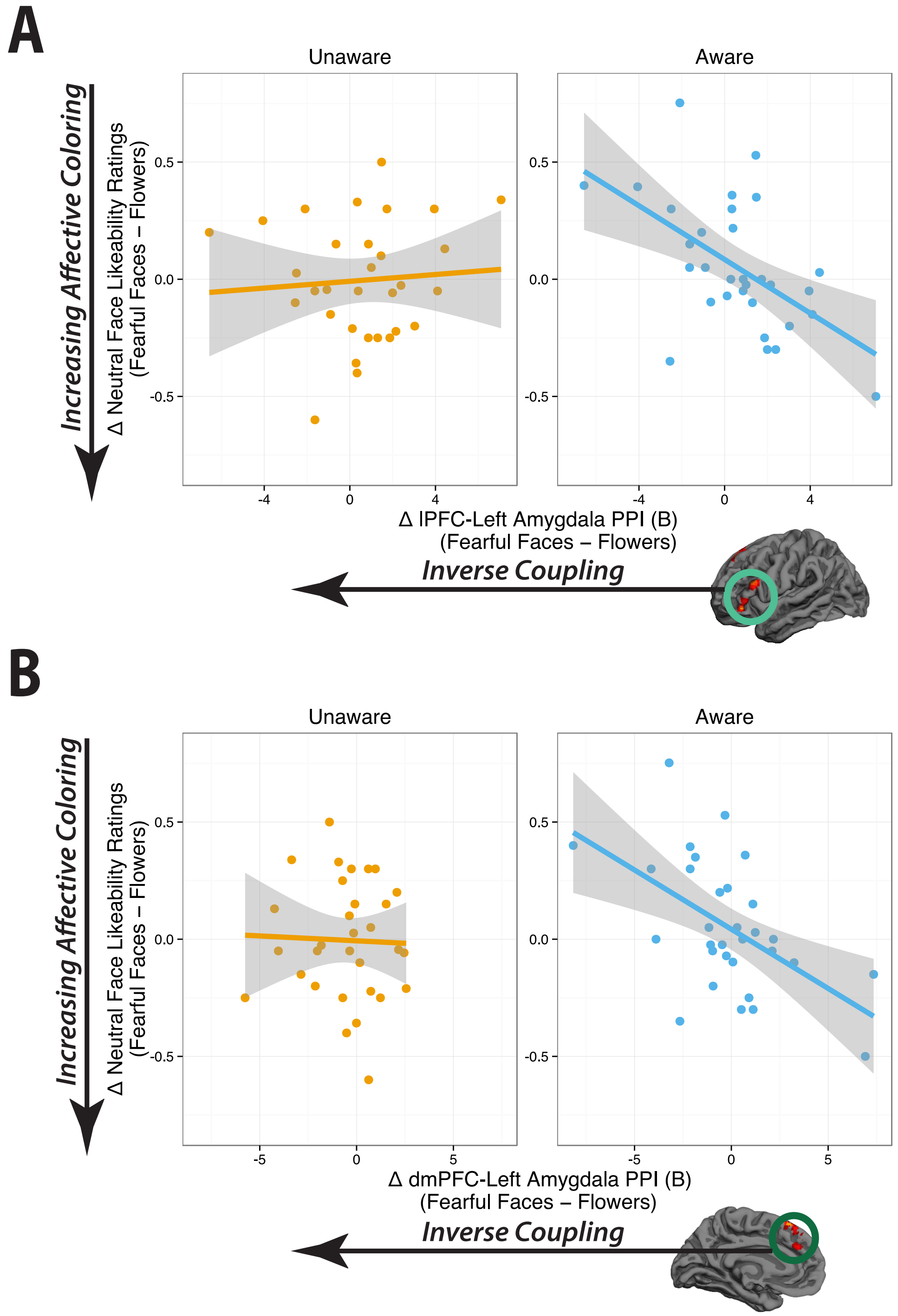

Supplementary Figure 2. When individuals were consciously aware of the stimuli, greater left uncinate fasciculus FA was associated with more inverse dorsomedial PFC-left amygdala coupling in response to fearful faces (relative to flowers), $p = .036$

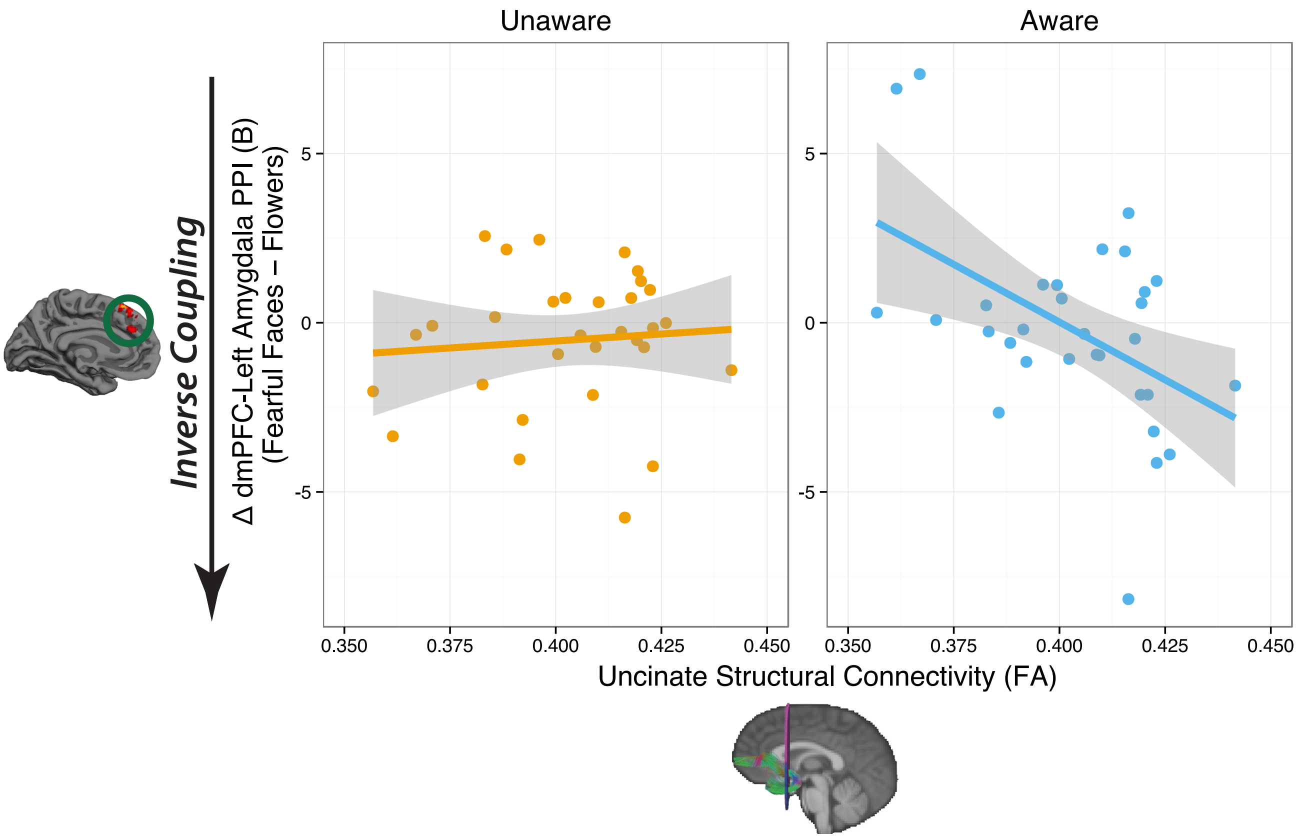

Supplement: Supplementary Information [file srep25826-s1.pdf]
